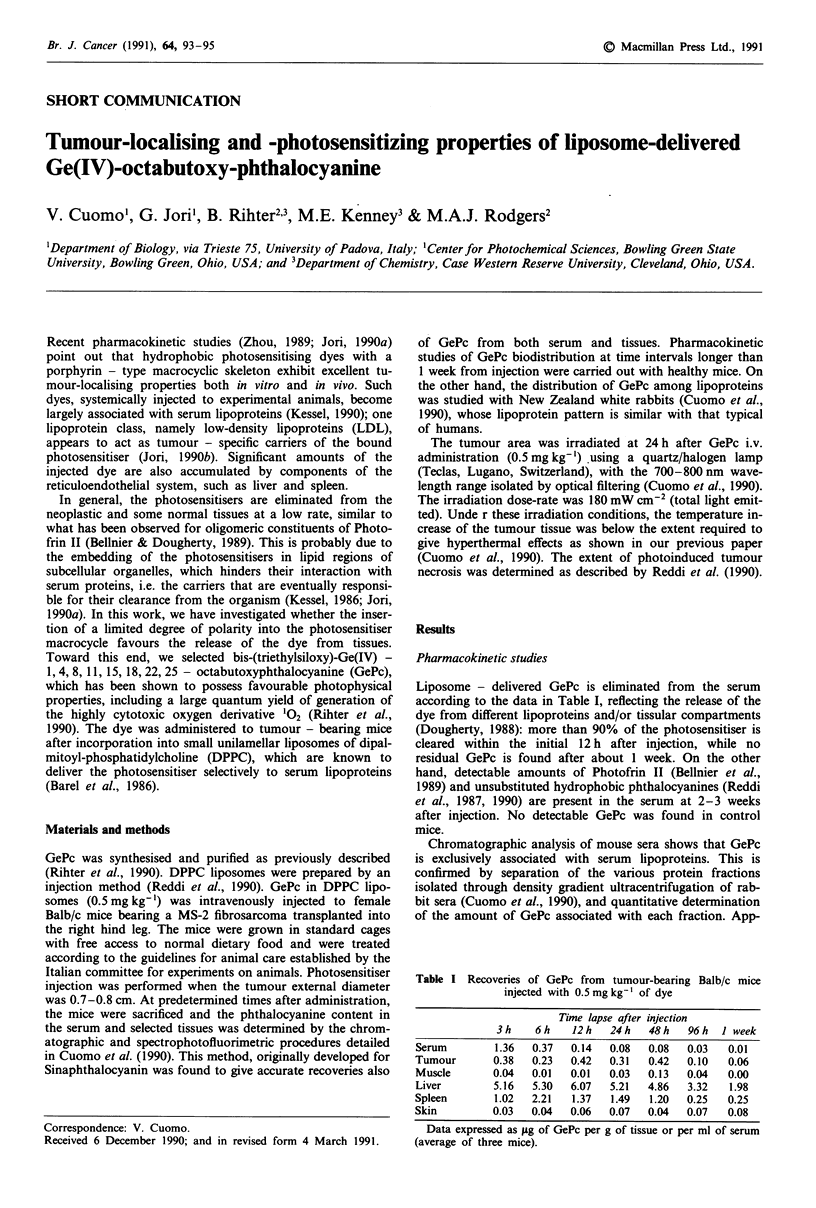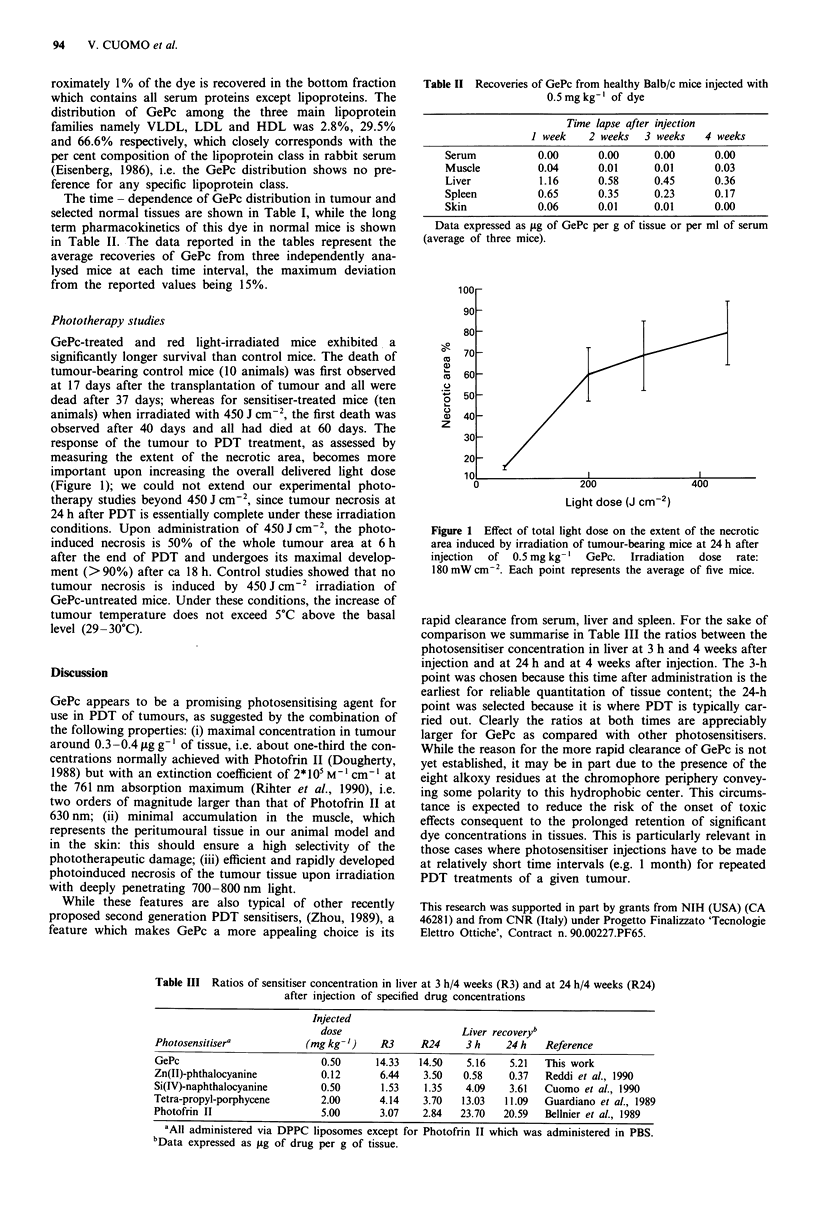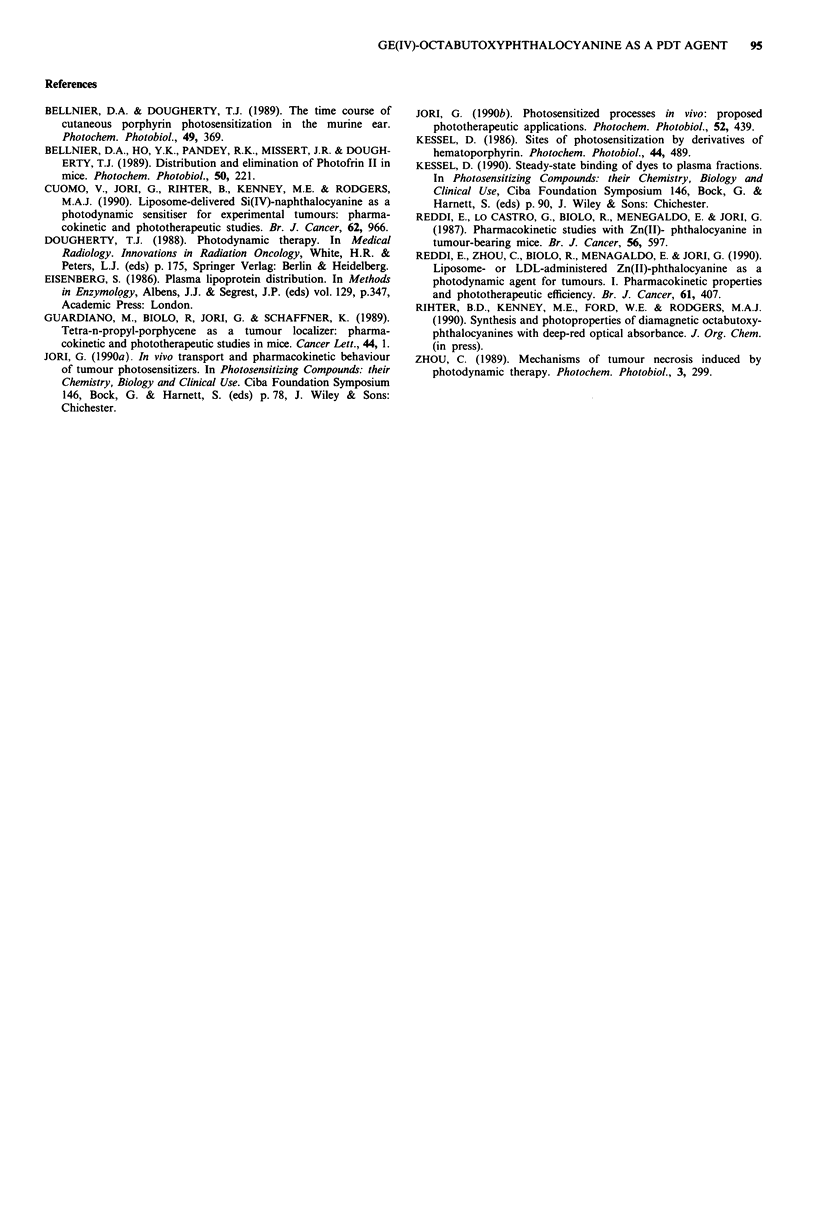# Tumour-localising and -photosensitizing properties of liposome-delivered Ge(IV)-octabutoxy-phthalocyanine.

**DOI:** 10.1038/bjc.1991.247

**Published:** 1991-07

**Authors:** V. Cuomo, G. Jori, B. Rihter, M. E. Kenney, M. A. Rodgers

**Affiliations:** Department of Biology, University of Padova, Italy.


Br. J. Cancer (1991), 64, 93-95

© Macmillan Press Ltd., 1991

SHORT COMMUNICATION

### Tumour-localising and -photosensitizing properties of liposome-delivered Ge(IV)-octabutoxy-phthalocyanine

V. Cuomo[1], G. Jori[1], B. Rihter[2,3], M.E. Kenney[3] & M.A.J. Rodgers[2]

[1]*Department of Biology, via Trieste 75, University of Padova, Italy;* [1]*Center for Photochemical Sciences, Bowling Green State University, Bowling Green, Ohio, USA; and* [3]*Department of Chemistry, Case Western Reserve University, Cleveland, Ohio, USA.*

Recent pharmacokinetic studies (Zhou, 1989; Jori, 1990*a*) point out that hydrophobic photosensitising dyes with a porphyrin – type macrocyclic skeleton exhibit excellent tumour-localising properties both *in vitro* and *in vivo*. Such dyes, systemically injected to experimental animals, become largely associated with serum lipoproteins (Kessel, 1990); one lipoprotein class, namely low-density lipoproteins (LDL), appears to act as tumour – specific carriers of the bound photosensitiser (Jori, 1990*b*). Significant amounts of the injected dye are also accumulated by components of the reticuloendothelial system, such as liver and spleen.

In general, the photosensitisers are eliminated from the neoplastic and some normal tissues at a low rate, similar to what has been observed for oligomeric constituents of Photofrin II (Bellnier & Dougherty, 1989). This is probably due to the embedding of the photosensitisers in lipid regions of subcellular organelles, which hinders their interaction with serum proteins, i.e. the carriers that are eventually responsible for their clearance from the organism (Kessel, 1986; Jori, 1990*a*). In this work, we have investigated whether the insertion of a limited degree of polarity into the photosensitiser macrocycle favours the release of the dye from tissues. Toward this end, we selected bis-(triethylsiloxy)-Ge(IV) – 1, 4, 8, 11, 15, 18, 22, 25 – octabutoxyphthalocyanine (GePc), which has been shown to possess favourable photophysical properties, including a large quantum yield of generation of the highly cytotoxic oxygen derivative $^1O_2$ (Rihter *et al.*, 1990). The dye was administered to tumour – bearing mice after incorporation into small unilamellar liposomes of dipalmitoyl-phosphatidylcholine (DPPC), which are known to deliver the photosensitiser selectively to serum lipoproteins (Barel *et al.*, 1986).

### Materials and methods

GePc was synthesised and purified as previously described (Rihter *et al.*, 1990). DPPC liposomes were prepared by an injection method (Reddi *et al.*, 1990). GePc in DPPC liposomes ($0.5\ mg\ kg^{-1}$) was intravenously injected to female Balb/c mice bearing a MS-2 fibrosarcoma transplanted into the right hind leg. The mice were grown in standard cages with free access to normal dietary food and were treated according to the guidelines for animal care established by the Italian committee for experiments on animals. Photosensitiser injection was performed when the tumour external diameter was 0.7–0.8 cm. At predetermined times after administration, the mice were sacrificed and the phthalocyanine content in the serum and selected tissues was determined by the chromatographic and spectrophotofluorimetric procedures detailed in Cuomo *et al.* (1990). This method, originally developed for Sinaphthalocyanin was found to give accurate recoveries also of GePc from both serum and tissues. Pharmacokinetic studies of GePc biodistribution at time intervals longer than 1 week from injection were carried out with healthy mice. On the other hand, the distribution of GePc among lipoproteins was studied with New Zealand white rabbits (Cuomo *et al.*, 1990), whose lipoprotein pattern is similar with that typical of humans.

The tumour area was irradiated at 24 h after GePc i.v. administration ($0.5\ mg\ kg^{-1}$) using a quartz/halogen lamp (Teclas, Lugano, Switzerland), with the 700–800 nm wavelength range isolated by optical filtering (Cuomo *et al.*, 1990). The irradiation dose-rate was $180\ mW\ cm^{-2}$ (total light emitted). Unde r these irradiation conditions, the temperature increase of the tumour tissue was below the extent required to give hyperthermal effects as shown in our previous paper (Cuomo *et al.*, 1990). The extent of photoinduced tumour necrosis was determined as described by Reddi *et al.* (1990).

### Results

### *Pharmacokinetic studies*

Liposome – delivered GePc is eliminated from the serum according to the data in Table I, reflecting the release of the dye from different lipoproteins and/or tissular compartments (Dougherty, 1988): more than 90% of the photosensitiser is cleared within the initial 12 h after injection, while no residual GePc is found after about 1 week. On the other hand, detectable amounts of Photofrin II (Bellnier *et al.*, 1989) and unsubstituted hydrophobic phthalocyanines (Reddi *et al.*, 1987, 1990) are present in the serum at 2–3 weeks after injection. No detectable GePc was found in control mice.

Chromatographic analysis of mouse sera shows that GePc is exclusively associated with serum lipoproteins. This is confirmed by separation of the various protein fractions isolated through density gradient ultracentrifugation of rabbit sera (Cuomo *et al.*, 1990), and quantitative determination of the amount of GePc associated with each fraction. App-

**Table I** Recoveries of GePc from tumour-bearing Balb/c mice injected with $0.5\ mg\ kg^{-1}$ of dye

| | *Time lapse after injection* | | | | | | |
|---|---|---|---|---|---|---|---|
| | *3 h* | *6 h* | *12 h* | *24 h* | *48 h* | *96 h* | *1 week* |
| Serum | 1.36 | 0.37 | 0.14 | 0.08 | 0.08 | 0.03 | 0.01 |
| Tumour | 0.38 | 0.23 | 0.42 | 0.31 | 0.42 | 0.10 | 0.06 |
| Muscle | 0.04 | 0.01 | 0.01 | 0.03 | 0.13 | 0.04 | 0.00 |
| Liver | 5.16 | 5.30 | 6.07 | 5.21 | 4.86 | 3.32 | 1.98 |
| Spleen | 1.02 | 2.21 | 1.37 | 1.49 | 1.20 | 0.25 | 0.25 |
| Skin | 0.03 | 0.04 | 0.06 | 0.07 | 0.04 | 0.07 | 0.08 |

Data expressed as μg of GePc per g of tissue or per ml of serum (average of three mice).

Correspondence: V. Cuomo.
Received 6 December 1990; and in revised form 4 March 1991.

roximately 1% of the dye is recovered in the bottom fraction which contains all serum proteins except lipoproteins. The distribution of GePc among the three main lipoprotein families namely VLDL, LDL and HDL was 2.8%, 29.5% and 66.6% respectively, which closely corresponds with the per cent composition of the lipoprotein class in rabbit serum (Eisenberg, 1986), i.e. the GePc distribution shows no preference for any specific lipoprotein class.

The time - dependence of GePc distribution in tumour and selected normal tissues are shown in Table I, while the long term pharmacokinetics of this dye in normal mice is shown in Table II. The data reported in the tables represent the average recoveries of GePc from three independently analysed mice at each time interval, the maximum deviation from the reported values being 15%.

*Phototherapy studies*

GePc-treated and red light-irradiated mice exhibited a significantly longer survival than control mice. The death of tumour-bearing control mice (10 animals) was first observed at 17 days after the transplantation of tumour and all were dead after 37 days; whereas for sensitiser-treated mice (ten animals) when irradiated with 450 J $cm^{-2}$, the first death was observed after 40 days and all had died at 60 days. The response of the tumour to PDT treatment, as assessed by measuring the extent of the necrotic area, becomes more important upon increasing the overall delivered light dose (Figure 1); we could not extend our experimental phototherapy studies beyond 450 J $cm^{-2}$, since tumour necrosis at 24 h after PDT is essentially complete under these irradiation conditions. Upon administration of 450 J $cm^{-2}$, the photoinduced necrosis is 50% of the whole tumour area at 6 h after the end of PDT and undergoes its maximal development (>90%) after ca 18 h. Control studies showed that no tumour necrosis is induced by 450 J $cm^{-2}$ irradiation of GePc-untreated mice. Under these conditions, the increase of tumour temperature does not exceed 5°C above the basal level (29-30°C).

**Table II** Recoveries of GePc from healthy Balb/c mice injected with 0.5 mg $kg^{-1}$ of dye

| | *Time lapse after injection* | | | |
|---|---|---|---|---|
| | *1 week* | *2 weeks* | *3 weeks* | *4 weeks* |
| Serum | 0.00 | 0.00 | 0.00 | 0.00 |
| Muscle | 0.04 | 0.01 | 0.01 | 0.03 |
| Liver | 1.16 | 0.58 | 0.45 | 0.36 |
| Spleen | 0.65 | 0.35 | 0.23 | 0.17 |
| Skin | 0.06 | 0.01 | 0.01 | 0.00 |

Data expressed as μg of GePc per g of tissue or per ml of serum (average of three mice).

**Figure 1** Effect of total light dose on the extent of the necrotic area induced by irradiation of tumour-bearing mice at 24 h after injection of 0.5 mg $kg^{-1}$ GePc. Irradiation dose rate: 180 mW $cm^{-2}$. Each point represents the average of five mice.

### Discussion

GePc appears to be a promising photosensitising agent for use in PDT of tumours, as suggested by the combination of the following properties: (i) maximal concentration in tumour around 0.3-0.4 μg $g^{-1}$ of tissue, i.e. about one-third the concentrations normally achieved with Photofrin II (Dougherty, 1988) but with an extinction coefficient of $2*10^5$ $M^{-1}$ $cm^{-1}$ at the 761 nm absorption maximum (Rihter *et al.*, 1990), i.e. two orders of magnitude larger than that of Photofrin II at 630 nm; (ii) minimal accumulation in the muscle, which represents the peritumoural tissue in our animal model and in the skin: this should ensure a high selectivity of the phototherapeutic damage; (iii) efficient and rapidly developed photoinduced necrosis of the tumour tissue upon irradiation with deeply penetrating 700-800 nm light.

While these features are also typical of other recently proposed second generation PDT sensitisers, (Zhou, 1989), a feature which makes GePc a more appealing choice is its rapid clearance from serum, liver and spleen. For the sake of comparison we summarise in Table III the ratios between the photosensitiser concentration in liver at 3 h and 4 weeks after injection and at 24 h and at 4 weeks after injection. The 3-h point was chosen because this time after administration is the earliest for reliable quantitation of tissue content; the 24-h point was selected because it is where PDT is typically carried out. Clearly the ratios at both times are appreciably larger for GePc as compared with other photosensitisers. While the reason for the more rapid clearance of GePc is not yet established, it may be in part due to the presence of the eight alkoxy residues at the chromophore periphery conveying some polarity to this hydrophobic center. This circumstance is expected to reduce the risk of the onset of toxic effects consequent to the prolonged retention of significant dye concentrations in tissues. This is particularly relevant in those cases where photosensitiser injections have to be made at relatively short time intervals (e.g. 1 month) for repeated PDT treatments of a given tumour.

This research was supported in part by grants from NIH (USA) (CA 46281) and from CNR (Italy) under Progetto Finalizzato 'Tecnologie Elettro Ottiche', Contract n. 90.00227.PF65.

**Table III** Ratios of sensitiser concentration in liver at 3 h/4 weeks (R3) and at 24 h/4 weeks (R24) after injection of specified drug concentrations

| *Photosensitiser*[a] | *Injected dose (mg $kg^{-1}$)* | *R3* | *R24* | *Liver recovery*[b] *3 h* | *Liver recovery*[b] *24 h* | *Reference* |
|---|---|---|---|---|---|---|
| GePc | 0.50 | 14.33 | 14.50 | 5.16 | 5.21 | This work |
| Zn(II)-phthalocyanine | 0.12 | 6.44 | 3.50 | 0.58 | 0.37 | Reddi *et al.*, 1990 |
| Si(IV)-naphthalocyanine | 0.50 | 1.53 | 1.35 | 4.09 | 3.61 | Cuomo *et al.*, 1990 |
| Tetra-propyl-porphycene | 2.00 | 4.14 | 3.70 | 13.03 | 11.09 | Guardiano *et al.*, 1989 |
| Photofrin II | 5.00 | 3.07 | 2.84 | 23.70 | 20.59 | Bellnier *et al.*, 1989 |

[a]All administered via DPPC liposomes except for Photofrin II which was administered in PBS.
[b]Data expressed as μg of drug per g of tissue.

### References

BELLNIER, D.A. & DOUGHERTY, T.J. (1989). The time course of cutaneous porphyrin photosensitization in the murine ear. *Photochem. Photobiol.*, **49**, 369.

BELLNIER, D.A., HO, Y.K., PANDEY, R.K., MISSERT, J.R. & DOUGHERTY, T.J. (1989). Distribution and elimination of Photofrin II in mice. *Photochem. Photobiol.*, **50**, 221.

CUOMO, V., JORI, G., RIHTER, B., KENNEY, M.E. & RODGERS, M.A.J. (1990). Liposome-delivered Si(IV)-naphthalocyanine as a photodynamic sensitiser for experimental tumours: pharmacokinetic and phototherapeutic studies. *Br. J. Cancer*, **62**, 966.

DOUGHERTY, T.J. (1988). Photodynamic therapy. In *Medical Radiology. Innovations in Radiation Oncology*, White, H.R. & Peters, L.J. (eds) p. 175, Springer Verlag: Berlin & Heidelberg.

EISENBERG, S. (1986). Plasma lipoprotein distribution. In *Methods in Enzymology*, Albens, J.J. & Segrest, J.P. (eds) vol. 129, p.347, Academic Press: London.

GUARDIANO, M., BIOLO, R, JORI, G. & SCHAFFNER, K. (1989). Tetra-n-propyl-porphycene as a tumour localizer: pharmacokinetic and phototherapeutic studies in mice. *Cancer Lett.*, **44**, 1.

JORI, G. (1990*a*). *In vivo* transport and pharmacokinetic behaviour of tumour photosensitizers. In *Photosensitizing Compounds: their Chemistry, Biology and Clinical Use*. Ciba Foundation Symposium 146, Bock, G. & Harnett, S. (eds) p. 78, J. Wiley & Sons: Chichester.

JORI, G. (1990*b*). Photosensitized processes *in vivo*: proposed phototherapeutic applications. *Photochem. Photobiol.*, **52**, 439.

KESSEL, D. (1986). Sites of photosensitization by derivatives of hematoporphyrin. *Photochem. Photobiol.*, **44**, 489.

KESSEL, D. (1990). Steady-state binding of dyes to plasma fractions. In *Photosensitizing Compounds: their Chemistry, Biology and Clinical Use*, Ciba Foundation Symposium 146, Bock, G. & Harnett, S. (eds) p. 90, J. Wiley & Sons: Chichester.

REDDI, E., LO CASTRO, G., BIOLO, R., MENEGALDO, E. & JORI, G. (1987). Pharmacokinetic studies with Zn(II)- phthalocyanine in tumour-bearing mice. *Br. J. Cancer*, **56**, 597.

REDDI, E., ZHOU, C., BIOLO, R., MENAGALDO, E. & JORI, G. (1990). Liposome- or LDL-administered Zn(II)-phthalocyanine as a photodynamic agent for tumours. I. Pharmacokinetic properties and phototherapeutic efficiency. *Br. J. Cancer*, **61**, 407.

RIHTER, B.D., KENNEY, M.E., FORD, W.E. & RODGERS, M.A.J. (1990). Synthesis and photoproperties of diamagnetic octabutoxyphthalocyanines with deep-red optical absorbance. *J. Org. Chem.* (in press).

ZHOU, C. (1989). Mechanisms of tumour necrosis induced by photodynamic therapy. *Photochem. Photobiol.*, **3**, 299.